# Detection of betacyanin in red-tube spinach (*Spinacia oleracea*) and its biofortification by strategic hydroponics

**DOI:** 10.1371/journal.pone.0203656

**Published:** 2018-09-07

**Authors:** Sho Watanabe, Yuta Ohtani, Wataru Aoki, Yuko Uno, Yasunori Sukekiyo, Seiichi Kubokawa, Mitsuyoshi Ueda

**Affiliations:** 1 Division of Applied Life Sciences, Graduate School of Agriculture, Kyoto University, Sakyo-ku, Kyoto, Japan; 2 Kyoto Integrated Science & Technology Bio- Analysis Center, Shimogyo-ku, Kyoto, Japan; 3 Mitsubishi Chemical, Chiyoda-ku, Tokyo, Japan; Huazhong University of Science and Technology, CHINA

## Abstract

Betacyanins have been reported as water-soluble, nitrogenous pigments found in the order *Caryophyllales*, and they are known for powerful natural antioxidant. The biofortification of secondary metabolites such as anthocyanins and betacyanins has recently been performed in food crops by metabolic engineering through genetic modification. However, the distribution of genetically modified foods is strictly regulated. Therefore, we aimed to develop a new method for biofortifying natural antioxidants, betacyanins, without genetic modification. We first detected the presence of betacyanins in red-tube spinach (*Spinacia oleracea*) through ultraviolet-visible spectroscopy and mass spectrometry. We then hydroponically cultivated plants in the presence of three candidate compounds for betacyanin biofortification: dopamine, Ca^2+^, and sucrose. Liquid chromatography–tandem mass spectrometry (LC–MS/MS) and antioxidant activity analyses showed that sucrose was most successful in biofortifying betacyanins, and reverse transcription polymerase chain reaction (RT-PCR) indicated that several genes involved in betacyanin biosynthesis were induced by sucrose. Therefore, strategic hydroponics represents a new approach for cultivating betacyanin-enriched vegetables.

## Introduction

**Fig 1:**
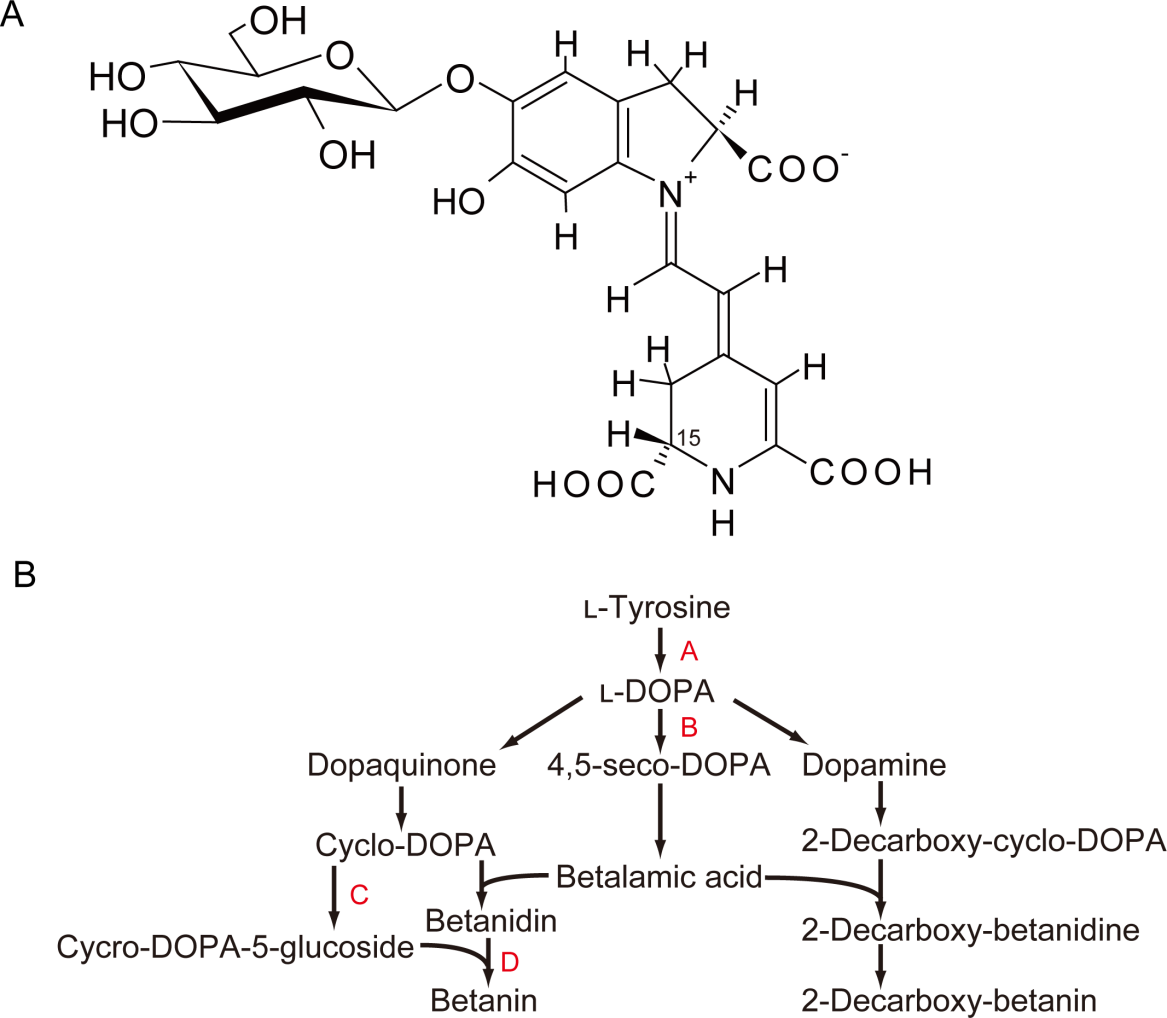
Characteristics of betacyanins. (A) The chemical structure of betanin, the most common betacyanin. Isobetanin is a structural isomer at the point marked “15.” (B) The biosynthesis pathway of betacyanins in plants showing the following enzyme steps: A, tyrosinase; B, 4,5-DOPA dioxygenase; C, cyclo-DOPA 5-glucoside transferase; D, 5-glucoside transferase.

Plant pigments are of interest to both biologists and food scientists [1]. Red colors in plants are mainly derived from two types of pigments: anthocyanins, which are broadly distributed among plants, and betacyanins, which are only found in plants in the order *Caryophyllales*, such as red beet (*Beta vulgaris* var. *cicla*) and amaranthus (*Amaranthus* spp.) [2,3]. Betacyanins are the general term for a class of pigments, and betanin is known as a principle one. Betacyanins are structurally different from anthocyanins, and are characterized by the inclusion of nitrogen in their chemical structure (Fig 1A). These pigments also have different biosynthesis pathways, with anthocyanins being synthesized from phenylalanine and betacyanins being synthesized from tyrosine (Fig 1B) [2].

Though the biological functions of anthocyanins are well known [2,4], both the biological functions and biosynthesis mechanisms of betacyanins remain poorly understood [5]. However, some studies have reported that betacyanins have stronger antioxidant activity than anthocyanins or other secondary metabolites that are generally known as strong antioxidants [6]. Moreover, it has recently been shown that betacyanins also have anticarcinogenic properties and are able to suppress malignant cell proliferation *in vitro* [7,8]. Consequently, their biological functions are receiving increasing attention.

The biofortification of red pigments in plants has recently been achieved by manipulating the biosynthetic process using two different approaches. In the first approach, biotic or abiotic elicitors are used to increase the production of anthocyanins or betacyanins. However, to date, this approach has only been successfully applied to plant cell cultures or callus cultures [9,10], with no report on its application in individual plants. The second approach involves genetic modification. For example, Tohge *et al*. successfully transformed the Delila (*Del*) and Rosea1 (*Ros1*) genes, which are known to elevate transcript levels of anthocyanin biosynthetic genes, from snapdragon (*Antirrhinum majus*) to tomato (*Solanum lycopersicum*) [11], resulting in the transformed tomatoes accumulating anthocyanins at levels that were substantially higher than wild-type tomatoes, which are unable to produce anthocyanins, and at comparable levels to those found in blackberries (*Rubus* spp.) and blueberries (*Cyanococcus* spp.) [12]. Furthermore, Harris *et al*. induced betacyanin production in potato by introducing DNA constructs to cause the transient production of DOPA 4,5-dioxygenase (DOD), which converts l-dopa to betalamic acid [13]. Betacyanin biofortification in tomato and potato (*Solanum tuberosum*) has also been accomplished by transforming and overexpressing several genes that are involved in the biosynthesis of betacyanins [14]. However, genetic modification techniques were used in all of these studies, the production and distribution of the resultant crops would be strictly regulated or restricted in many countries [15].

In a previous report, we constructed a hydroponic cultivation system to develop nutritious vegetables by adjusting the composition of liquid fertilizer [16]. In that study, we successfully produced biofortified spinach with a two-fold greater content of folate by hydroponically cultivating spinach plants in a nutrient solution containing phenylalanine [16]. This approach has the advantages of not involving genetic modification, meaning that circulation of the resultant biofortified vegetables will not be restricted in the market, as well as being easy to perform at a reasonable cost through the use of inexpensive compounds.

In this study, we detected the production of betacyanins in red-tube spinach (*Spinacia orelacea* L.) and aimed to develop a novel method for biofortifying the betacyanin content of red-tube spinach (*Spinacia orelacea* L.) through hydroponic cultivation to examine the broader applicability of our recently developed biofortification method [16] and to improve our understanding of the mechanism of betacyanin biosynthesis.

## Results

### Identification of betacyanins

**Fig 2:**
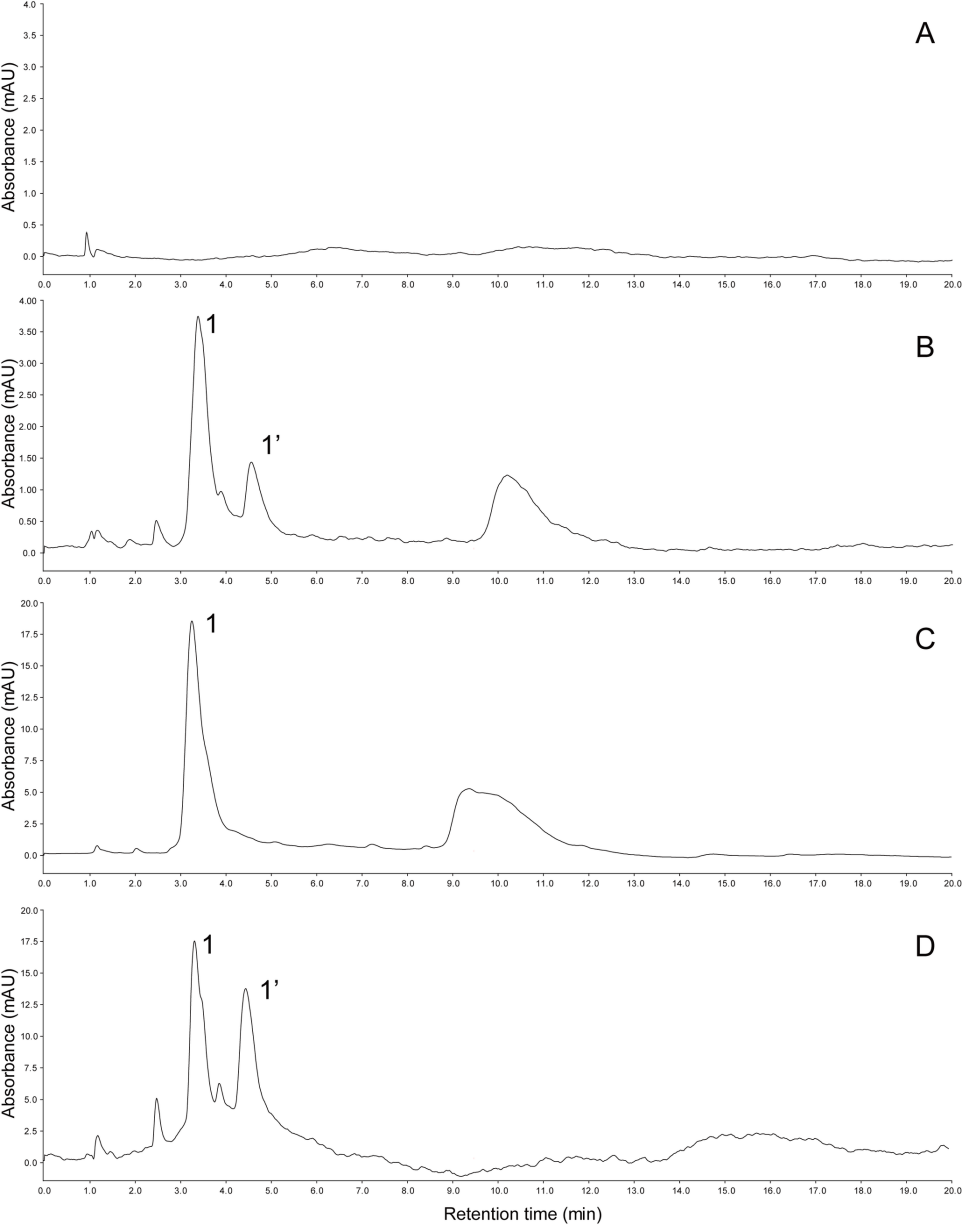
High-performance liquid chromatography (HPLC) chromatograms of (A) the green-tube spinach (*Spinacia oleracea*) strain NPL08, (B) the red-tube spinach strain Sosei salad akari, (C) the red-tube spinach strain Banchu akakuki minster, and (D) red beet (*Beta vulgaris*) extract (positive control) monitored at 538 nm. Possible betacyanins (1 and 1’) were identified using mass spectrometry (see Table 1).

Since spinach belongs to the order Caryophylalles, it has been assumed that the red pigments that occur in red-tube varieties are betacyanins [3]. However, there was no report showing that red pigments in *S*. *orelacea* is derived from betacyanins. Therefore, pigments were extracted from two strains of red-tube spinach (Sosei salad akari and Banchu akakuki minster) as well as one strain of green-tube spinach (NPL08) as a control (S1 Fig). It is known that betacyanins have a characteristic maximum absorption peak at a wavelength of 538 nm [17]. No characteristic peak was found at 538 nm in the green-tube spinach, whereas multiple peaks derived from betacyanins were detected at 538 nm in both red-tube spinach strains as well as the red beet extract that was used as a positive control (Fig 2). Mass spectra was shown in S2 Fig.

**Table 1:** Betacyanins contained in red-tube spinach (*Spinacia oleracea*).

no.^a^	compound^b^	retention time (min)	*m/z* (M+H^+^)
1	betanin	3.4	551
1’	isobetanin	4.5	551

^*a*^ These numbers correspond to the peaks shown in Fig 2.

^*b*^ Each compound was identified based on its retention time and *m/z* [18].

### Enrichment of betacyanins in red-tube spinach

To investigate whether the betacyanin content of red-tube spinach could be biofortified, three candidate compounds that were expected to increase the amount of betacyanins were added to the hydroponic culture system: dopamine, which is a precursor of 2-decarboxybetanin (Fig 1B) [18] and the supply of which increases the demand for betalamic acid, resulting in activation of 2-decarboxybetanin or betanin synthesis [19]; calcium ions (Ca^2+^), which are known to be the best abiotic elicitor for increasing betacyanin contents [10]; and sucrose, which is known to be the best carbohydrate for secondary metabolite production [20]. We determined the doses of compounds based on these previous studies, and the biofortification schema on our previous study [16].

### Biofortification of betanin in red-tube spinach

**Fig 3:**
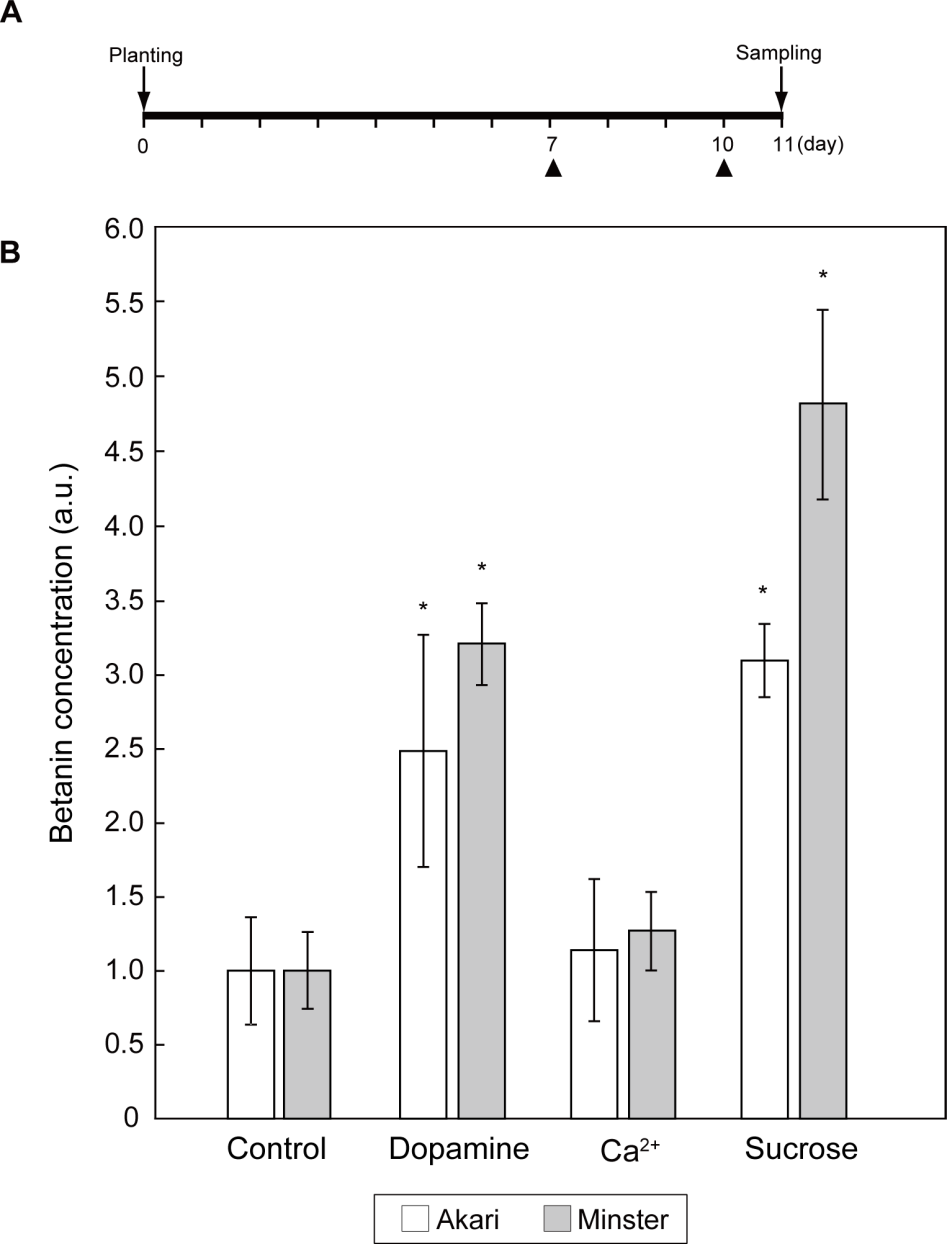
Biofortification of betanin. (A) Cultivation scheme for the compound-added spinach (*Spinacia oleracea*). Triangles indicate the addition of each compound. In the dopamine-added treatment, 2 mM dopamine was added twice to give a final concentration of 4 mM; in the Ca^2+^-added treatment, 1 mM calcium lactate was added twice to give a final concentration of 2 mM; and in the sucrose-added treatment, 10 mM sucrose was added twice to give a final concentration of 20 mM. (B) Relative betanin concentration in the spinach samples. Bars indicate the means ± standard deviations of three biological replicates. The statistical significance of differences between the compound-added samples and the control samples (no addition of compounds) was determined by Dunnett’s test (* *p* < 0.05). Control means each red-tube spinach without addition of compounds. Akari, Sosei salad akari; Minster, Banchu akakuki minster.

Red-tube spinach plants were hydroponically cultivated in liquid fertilizer supplemented with three candidate compounds that were expected to enhance betacyanin production (Fig 3A). Spinach cultivated in the Ca^2+^-added treatments showed similar growth to the control samples, whereas spinach cultivated in the dopamine- or sucrose-added treatments exhibited slightly lower growth than the control samples (Table 2).

**Table 2:** Growth of red-tube spinach (*Spinacia oleracea*) in different compound-added solutions.

no.	added compound	strain	plant length (mm)	fresh weight (g)
1	no addition	Akari	188 ± 5.9	7.5 ± 1.0
		Minster	158 ± 1.2	7.5 ± 1.1
2	dopamine	Akari	132 ± 5.1*	3.6 ± 0.4*
		Minster	118 ± 4.2*	3.3 ± 0.6*
3	Ca^2+^	Akari	190 ± 15.4	8.7 ± 1.2
		Minster	159 ± 5.0	8.0 ± 0.3
4	sucrose	Akari	144 ± 11.2*	4.6 ± 0.4*
		Minster	127 ± 2.9*	4.2 ± 0.4*

Samples were cultured following the schedule shown in Fig 2A. Data represent the means ± standard deviations of three biological replicates. The statistical significance of differences between the compound-added samples and the control samples (no addition of compounds) was determined by Dunnett’s test (* *p* < 0.05). Akari, Sosei salad akari; Minster, Banchu akakuki minster.

The betanin contents of the dopamine- and sucrose-added samples were significantly increased by 3.2- and 4.8-fold, respectively, compared with the control samples, whereas that of the Ca^2+^-added samples remained unchanged (Fig 3B).

### Antioxidant activity of biofortified red-tube spinach

**Fig 4:**
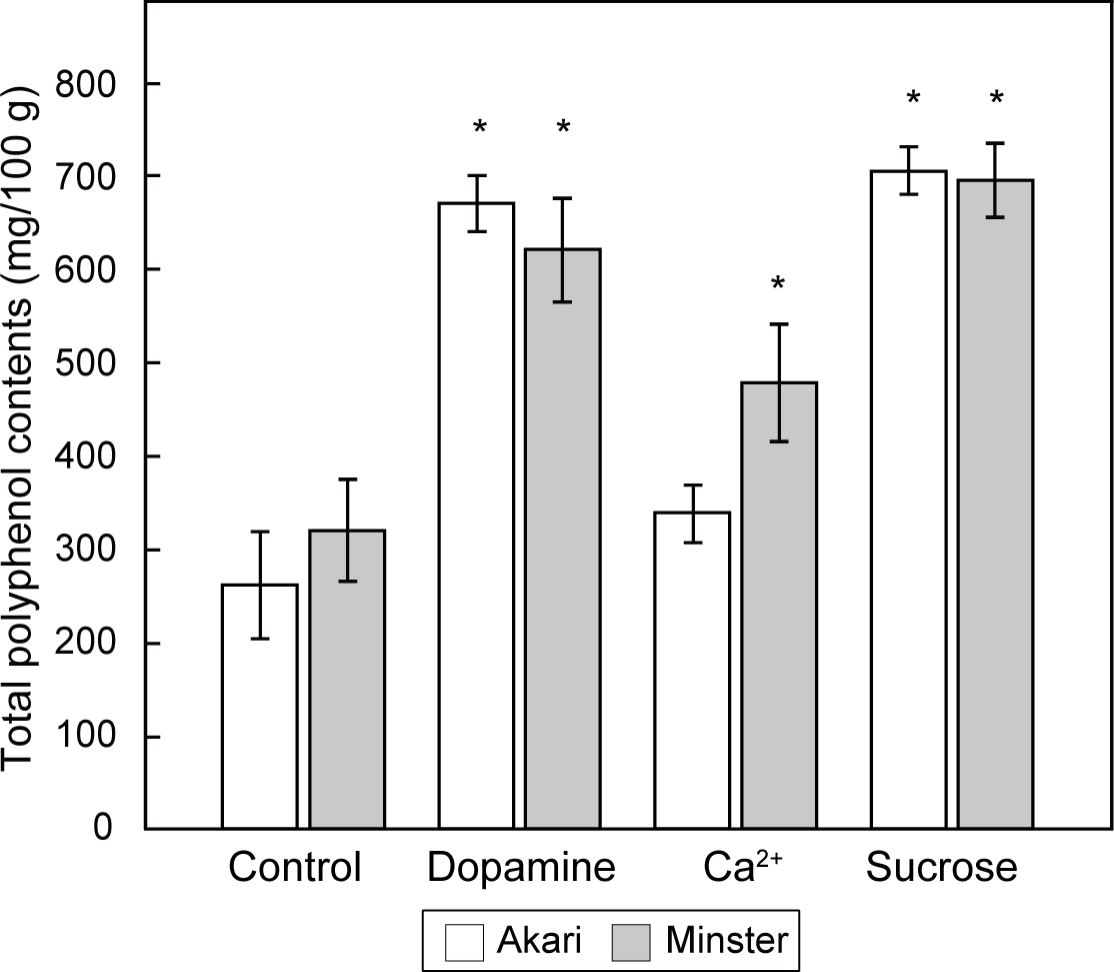
Assessment of the antioxidant activity of red-tube spinach (*Spinacia oleracea*) grown in different compound-added solutions. Two strains of red-tube spinach were cultured in dopamine-, Ca^2+^-, and sucrose-added solutions following the schedule shown in Fig 2A. Bars represent the means ± standard deviations of three biological replicates. The statistical significance of differences between the compound-added samples and the control samples (no addition of compounds) was determined by Dunnett’s test (* *p* < 0.05). Akari, Sosei salad akari; Minster, Banchu akakuki minster.

**Fig 5:**
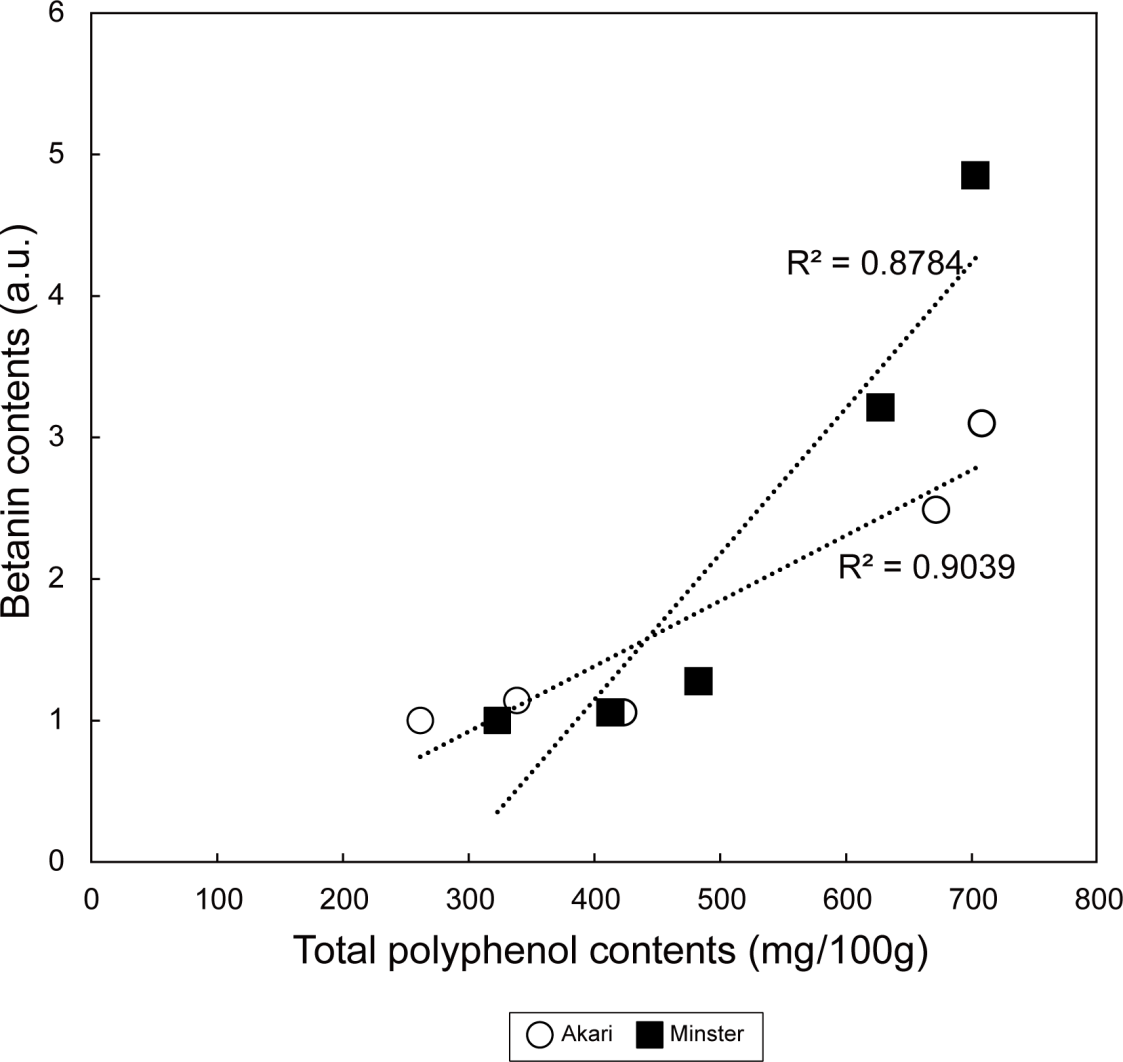
Correlation between the betanin content and antioxidant activity of two strains of red-tube spinach. Akari, Sosei salad akari; Minster, Banchu akakuki minster.

Since betacyanins are known to be powerful antioxidants [6], the antioxidant activity of the biofortified spinach was evaluated using the Folin-Ciocalteu method, which quantifies the total polyphenol contents. The dopamine- and sucrose-added samples exhibited significantly higher levels of antioxidants than the control samples for both strains of red-tube spinach, whereas in the case of the Ca^2+^-added samples, only one strain (Banchu akakuki minster) had significantly higher levels (Fig 4). There was a correlation between the betanin content and antioxidant activity (Fig 5). We measured antioxidant activity by two additional methods: Oxygen Radical Antioxidant Capacity (ORAC) assay and Trolox Equivalent Antioxidant Capacity (TEAC) assay. As a result, we confirmed similar increasing tendency of antioxidant activity of the biofortified red-tube spinach (S3 Fig).

### Quantification of transcript levels by real-time PCR

**Fig 6:**
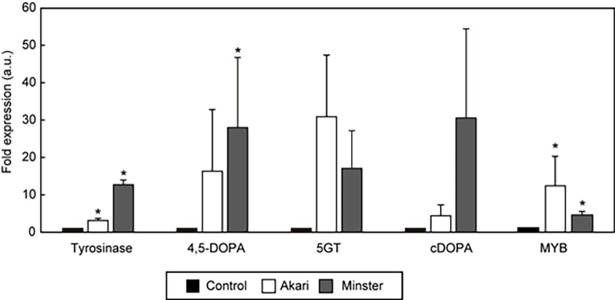
Relative expression levels of genes involved in betacyanin biosynthesis in two strains of red-tube spinach (*Spinacia oleracea*) grown in sucrose-added solution. The fold-change values are relative to control samples with no addition of sucrose (expression level = 1). Error bars indicate the standard errors from three independent experiments. The statistical significance of differences between the sucrose-added samples and the control samples was determined by Student’s *t*-test (* *p* < 0.05). Tyrosinase (enzyme A in Fig 1B), tyrosinase gene; 4,5-DOPA (enzyme B in Fig 1B), 4,5-DOPA dioxygenase gene; 5GT (enzyme D in Fig 1B), 5-glucoside transferase gene; cDOPA (enzyme C in Fig 1B), cyclo-DOPA gene; MYB, MYB (myeloblastosis protein) family protein gene; Akari, Sosei salad akari; Minster, Banchu akakuki minster.

To clarify the mechanism underlying the effect of sucrose on betacyanin synthesis, the relative transcript levels of four target genes that encode enzymes involved in betacyanin biosynthesis (Fig 1B: genes encoding enzymes A–D) were quantified using RT-PCR. Sucrose had a tendency to increase the levels of all of these genes, suggesting that it activated the metabolic pathway for betacyanin synthesis (Fig 6).

## Discussion

Betacyanins have never been reported to co-occur with anthocyanins [21] and are only found in plants in the order *Caryophyllales* [1], such as red beet and love-lies-bleeding (*Amaranthus caudatus*). In the phylogenic view, it has been assumed that the red pigments that occur in red-tube spinach, which belong to the *Caryophyllales*, are derived from betacyanins. However, identification of red pigments in red-tube spinach has not been reported. According to the UV-vis spectra and MS data, we found that red-tube spinach contains several betacyanins (Fig 2B), with the largest peak being derived from betanin. Similarly, betacyanins have been reported in red beet and amaranthus [18,22], among which betanin is also the most abundant [23].

**Fig 7:**
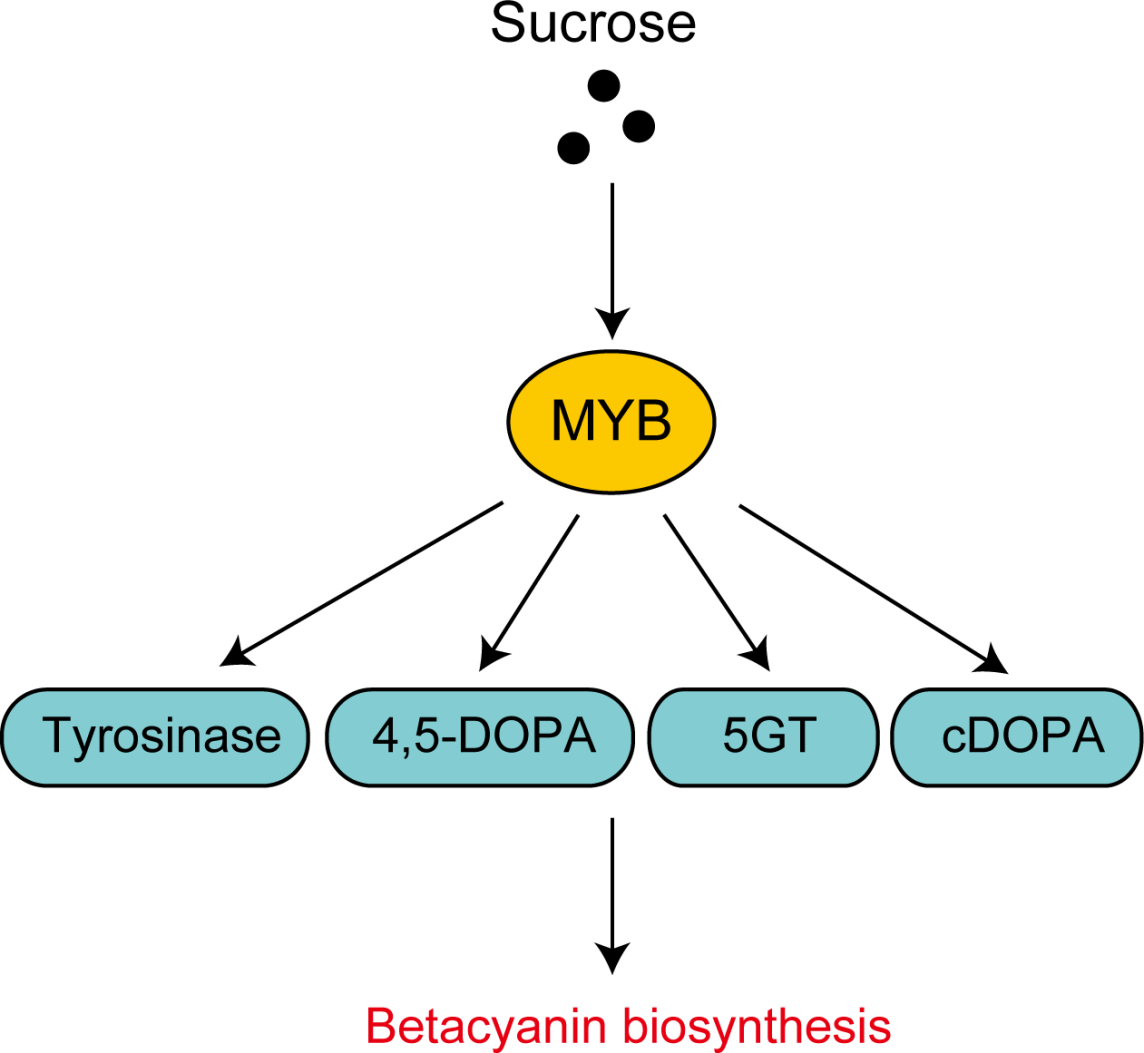
Possible pathway by which sucrose biofortifies red-tube spinach (*Spinacia oleracea*) via MYB transcription factors. Each arrow indicates the activation of a particular gene or reaction. The yellow ellipse indicates the transcription factor and blue ellipses indicate the genes involved in betacyanin synthesis. Tyrosinase, tyrosinase gene; 4,5-DOPA, 4,5-DOPA dioxygenase gene; 5GT, 5-O-glucoside transferase gene; cDOPA, cyclo-DOPA gene, MYB, MYB family protein.

The hydroponic cultivation of red-tube spinach in compound-added solutions showed that the addition of both dopamine and sucrose significantly increased the betanin content (Fig 3). In addition, Folin-Ciocalteu analysis showed that the total polyphenol contents of the spinach plants increased in the presence of all three additives (Fig 4). The effect of sucrose addition on the betanin content is thought to be complex, with two potential mechanisms being involved. First, it is possible that sucrose acts as an endogenous trigger, modulating the expression of betacyanin biosynthetic genes. It has previously been shown that plants grown on a sucrose-containing medium exhibit high levels of secondary metabolites such as anthocyanins [24], and it has been suggested that several genes involved in the biosynthesis of anthocyanins are induced by sucrose [25,26]. Similarly, in the present study, real-time PCR showed that some of the genes that are involved in betacyanin biosynthesis were induced by sucrose (Fig 6), including the myeloblastosis protein (MYB) gene. MYB is known to be a transcriptional regulator that is involved in the biosynthesis of flavonoid pigments in plants [27]. Furthermore, the sucrose-specific induction of anthocyanin biosynthesis via MYB regulators has been observed in *Arabidopsis* [26] and Lloyd *et al*. suggested that an anthocyanin-regulating MYB could also regulate the betalain pigment pathway [28], with three enzymatic steps associated with betacyanin biosynthesis potentially being regulated by MYB. Then, we inferred that activation of the MYB transcription factor was induced, and several enzymatic steps were activated by the addition of sucrose, resulting in increased betacyanin biosynthesis (Fig 7). However we don’t exclude the involvements of other transcription factors, and further research will be necessary. Second, it is possible that betacyanins are produced during intensive cell division [29]. Previous studies using suspension cultures have suggested that plant growth regulators and nutritional factors affect not only growth but also the production of secondary metabolites [30], and it has also been shown that there is a positive correlation between betacyanin accumulation and cell division [31].

Dopamine also increased the betacyanin content of red-tube spinach. Dopamine is a precursor of 2-decarboxybetanin which is synthesized from 3,4-dihydroxyphenylalanine (DOPA) and tyrosine (Fig 1B). Kobayashi *et al*. showed that the administration of dopamine activates tyrosinase and the production of 2-decarboxybetanin and betanin [19]. Moreover, betalamic acid, which is a precursor of most betacyanins, is required for 2-decarboxybetanin synthesis (Fig 1B). Therefore, it seems likely that dopamine administration increased the amount of betalamic acid, which was then used for the synthesis of other betacyanins such as betanin.

By contrast, the Ca^2+^-added solution had no effect on the betanin contents of red-tube spinach. Previous reports have shown that Ca^2+^ acts as an abiotic elicitor for increasing the production of secondary metabolites such as betacyanins [32]. However, it is possible that the amount of Ca^2+^ that was used in the present study was insufficient to increase the production of betacyanins. Therefore, further research is required to determine the optimal concentration that will enhance betacyanin production without causing a growth disorder. In addition, further research will be important to evaluate other potential elicitors such as Cu^2+^ which is contained in tyrosinase.

Red-tube spinach plants that were cultivated in sucrose- and dopamine-added solutions exhibited reduced growth compared with control plants (Table 2). White turbidity was observed in the sucrose-added fertilizer (data not shown), which was thought to have resulted from the growth of microorganisms. Therefore, it is possible that these microorganisms may have used the sucrose as a carbon source, leading to lower growth of the spinach. In terms of dopamine, catecholamines such as dopamine and epinephrine have been shown to be toxic to callus cultures of tobacco (*Nicotiana tabacum*) and three other plant species due to the regulation of cytokinin activity in the plant cells [33]. However, Protacio *et al*. suggested that the addition of catecholamines at low concentrations greatly stimulates the growth of tobacco [34]. Therefore, it may be possible to prevent growth disorders by lowering the concentration of sucrose and dopamine in the solution, indicating that additional experiments will be required to determine the optimal concentrations of these additives. In addition, octcloth, a silver-coated cloth pesticide, could be added to the nutrient solution tank to restrict the growth of microorganisms [16].

In this study, it was demonstrated for the first time that betacyanins occur in red-tube spinach and can be biofortified in hydroponic culture by manipulating the composition of the liquid fertilizer. We believe that this approach could be applied to many types of crops that can be cultivated hydroponically, greatly contributing to the development of additional functional foods.

## Materials and methods

### Reagents

Betanin (red beet extract diluted with dextrin) (Product number, P.N. CDS000584) was purchased from Sigma-Aldrich (St. Louis, MO, USA). 3,4-Dihydroxyphenethylamine hydrochloride (P.N. 62317), sucrose (P.N. 57501), sodium carbonate (P.N. 497198), methanol (P.N. 67561), acetonitrile (P.N. 75058), formic acid (P.N. 64186), and ultrapure water (P.N. 7732185) were purchased from Wako Pure Chemical Industries (Osaka, Japan). Calcium lactate for the Ca^2+^-added treatments (P.N. 814802) and trifluoroacetic acid (TFA) (P.N. 350709) were purchased from Nacalai Tesque Inc. (Kyoto, Japan). Folin-Ciocalteu phenol reagent (P.N. 02195186) was purchased from MP Biomedicals (Santa Ana, CA, USA). High-TEMPO Ar and High-TEMPO Cu were developed for liquid fertilizer by Mitsubishi Plastics Agri Dream Co. (Tokyo, Japan), as described previously [16].

### Hydroponic cultivation

The green-tube spinach strain NPL08 (Mitsubishi Plastics Agri Dream Co.) and the red-tube spinach strains Sosei salad akari (TAKII & Co., Ltd., Kyoto, Japan) and Banchu akakuki minster (Nakahara Seed Product Co., Ltd., Fukuoka, Japan) were used in this study (S1 Fig). All spinach samples were cultivated using the indoor hydroponic cultivation system Napperland® (Mitsubishi Plastics Agri Dream Co.), as described previously [16]. In brief, spinach seeds were germinated and grown for 3 days on granular rock wool, following which the young seedlings were grown for a further 11 days at 22°C under artificial light. The seedlings were then planted in a hydroponic cultivation system in which the liquid temperature was set to 20°C and the fertilizer was replaced 2 days before harvesting. The protocols that were used in the compound-added cultivation experiments are described in the Results section. Spinach samples were harvested at around noon on the 11th day and the fresh weight was measured. The samples were then stored at −80°C. Changes in pH during the hydroponic cultivation are described in the S1 Table.

### Extraction of metabolites for betacyanin identification

For identification of betacyanins by LC–MS, metabolites were extracted from each sample by grinding 5.0 g of stem to a fine powder in liquid nitrogen, transferring the powder to a 200-mL flask, and adding 100 mL of ultrapure water. Extraction was then facilitated by vortexing and sonicating the sample five times for 30 sec using a BIORUPTOR UCD-250 (Cosmo Bio). Following this, the sample was transferred to a 50-mL tube and centrifuged at 4°C and 8,000 × *g* for 20 min. The supernatant was then purified and fractionated on a C18 cartridge (GL Sciences, Kyoto, Japan) according to the following procedure. The C18 cartridge was first activated using 3 mL of 90% methanol with 0.1% TFA and 3 mL of 50% methanol with 0.1% TFA, and was then rinsed with 3 mL of 2% methanol with 0.1% TFA. The samples were applied to the column and rinsed again with 2% methanol with 0.1% TFA. The betacyanin fraction was then eluted with 70% methanol with 0.1% TFA and evaporated under reduced pressure. The purified betacyanin sample was dissolved in 200 μL of ultrapure water and analyzed by liquid chromatography–mass spectrometry (LC–MS) with an ultraviolet-visible (UV-vis) detector.

### Extraction and quantification of betanin

To quantify the betanin content and antioxidant activity of each sample, 100 mg of stem was ground to a fine powder in liquid nitrogen and transferred to a 1.5-mL tube to which 1.0 mL of ultrapure water was added. Extraction was then facilitated by vortexing and sonicating the sample five times for 30 sec using a BIORUPTOR UCD-250 (Cosmo Bio). The resultant solution was centrifuged at 4°C and 14,000 × *g* for 20 min, and the supernatant was analyzed by liquid chromatography–tandem mass spectrometry (LC–MS/MS) or using the Folin-Ciocalteu method.

### LC–MS analysis

Betacyanins were identified by LC (Nexera system; Shimadzu, Kyoto, Japan)–triple quadrupole mass spectrometry (LCMS-8060; Shimadzu). Samples (5 μL) were injected into an InertCore C18 column (150 mm × 2.1 mm I.D., 2.4 μm particle size; GL Sciences) at a flow rate of 400 μL/min. A gradient was produced by changing the mixing ratio of the two eluents: A, 0.1% (v/v) formic acid; and B, acetonitrile containing 0.1% (v/v) formic acid. The gradient was started with 5% B with a 4-min hold; this was then increased to 30% B for 30 min and then increased immediately to 100% B with a 3-min hold, following which the mobile phase was immediately adjusted to its initial composition and held for 4 min to re-equilibrate the column. The column temperature was set at 40°C. The autosampler (kept at 4°C) was equipped with a black door to prevent the samples from being exposed to light. Data acquisition for the estimation of betacyanins was performed at λ = 538 nm with a UV-vis high-performance liquid chromatography (HPLC) detector coupled with positive ion electrospray ionization LC–MS analysis (electrospray voltage, 4.0 kV; capillary temperature, 300°C; sheath gas, N_2_, 10 L/min).

### Multiple reaction monitoring analysis by LC–MS/MS

Betanin was quantified using the multiple reaction monitoring (MRM) mode by LC (Nexera system; Shimadzu, Kyoto, Japan)–triple quadrupole mass spectrometry (LCMS-8060; Shimadzu). Each parameters were shown below: precursor ion (*m/z*), 551.05; product ion (*m/z*), 389.20; Q1 pre bias, −30 V; Q3 pre bias, −27 V; collision energy, −29 V. Under the LC conditions described above, the retention time of betanin was 3.4 min and betanin showed much better sensitivity in positive mode than in negative mode.

### Assessment of antioxidant activity

The Folin-Ciocalteu assay is widely used to assess the total polyphenol content of samples [35]. This assay relies on the transfer of electrons from phenolic compounds to phosphomolybdic/phosphotungstic acid complexes in an alkaline medium to form blue complexes that are detected spectroscopically at 700 nm. This assay was used to assess the antioxidant activity of the spinach samples by adding 200 μL of each extracted sample to 500 μL of Folin-Ciocalteu reagent and leaving it for 5 min at room temperature. Following this, 500 μL of 10% sodium carbonate solvent in ultrapure water was added to each sample, mixed by vortex, and left for 1 h at room temperature. Color development was then determined at 700 nm using a spectrophotometer (UV-1700 PharmaSpec; Shimadzu). We measured antioxidant activity by two additional methods: Oxygen Radical Antioxidant Capacity (ORAC) assay (Cell BIOLABS, INC, CA, USA) and Trolox Equivalent Antioxidant Capacity (TEAC) assay (Cayman Chemical Company, MI, USA). In ORAC assay, samples and standards were measured with a fluorescent microplate reader (Fluoroskan Ascent FL, Thermo Fisher Scientific, USA) at 37°C with an excitation wavelength of 480 nm and an emission wavelength of 520 nm. In TEAC assay, color development measured at 405 nm using a spectrophotometer (UV-1700 PharmaSpec, Shimadzu).

### Isolation of RNA and preparation of cDNA

Total RNA was extracted from each spinach sample using the RNeasy Plant Mini Kit (QIAGEN, Hilden, Germany), according to the manufacturer’s protocol. cDNA was prepared using the High-Capacity cDNA Reverse Transcription Kit with genomic DNA remover (Applied Biosystems, Carlsbad, CA, USA). The reaction was performed according to the manufacturer’s protocol using 500 ng of total RNA as a template.

### Quantitative reverse transcription polymerase chain reaction (RT-PCR) analysis

Quantitative RT-PCR was performed using the Power SYBR^®^ Green PCR Master Mix (Applied Biosystems) in the 7500 Real-Time PCR System (Applied Biosystems). The glyceraldehyde-3-phosphate dehydrogenase (*GAPDH*) gene was used as an endogenous control to normalize the expression data for each gene and the primers were designed using the GenScript Real-time PCR (TaqMan) Primer Design tool (GenScript, New Jersey, USA) (see S2 Table). Sequences of the target genes were obtained from SpinachBase (http://www.spinachbase.org/cgi-bin/spinach/genome/search.cgi) [36].

## Supporting information

S1 Fig Strains of green- and red-tube spinach (*Spinacia oleracea*) used in this research. (A) NPL08; (B) Sosei salad akari; (C) Banshu akakuki minster.
(DOCX)

S2 Fig Mass spectra of betanin (m/z: 551.15) obtained by LC–MS. (DOCX)

S3 Fig Assessment of the antioxidant activity of red-tube spinach (*Spinacia oleracea*) grown in different compound-added solutions. Red-tube spinach were cultured in dopamine-, Ca^2+^-, and sucrose-added solutions following the schedule shown in Fig 2A. Bars represent the means ± standard deviations of three biological replicates. The statistical significance of differences between the compound-added samples and the control samples (no addition of compounds) was determined by Dunnett’s test (* p < 0.05). Minster, Banchu akakuki minster.
(DOCX)

S1 Table pH of the fertilizer during the hydroponic cultivation. (DOCX)

S2 Table Primers used in this study. (DOCX)
