# Supplementary material for: Detection of betacyanin in red-tube spinach (Spinacia oleracea) and its biofortification by strategic hydroponics
Source: PLoS One. 2018 Sep 7;13(9):e0203656. doi: 10.1371/journal.pone.0203656 (PMC6128657; doi:10.1371/journal.pone.0203656)
Supplement: S1 Fig — (A) NPL08; (B) Sosei salad akari; (C) Banshu akakuki minster. (DOCX) [file pone.0203656.s001.docx]

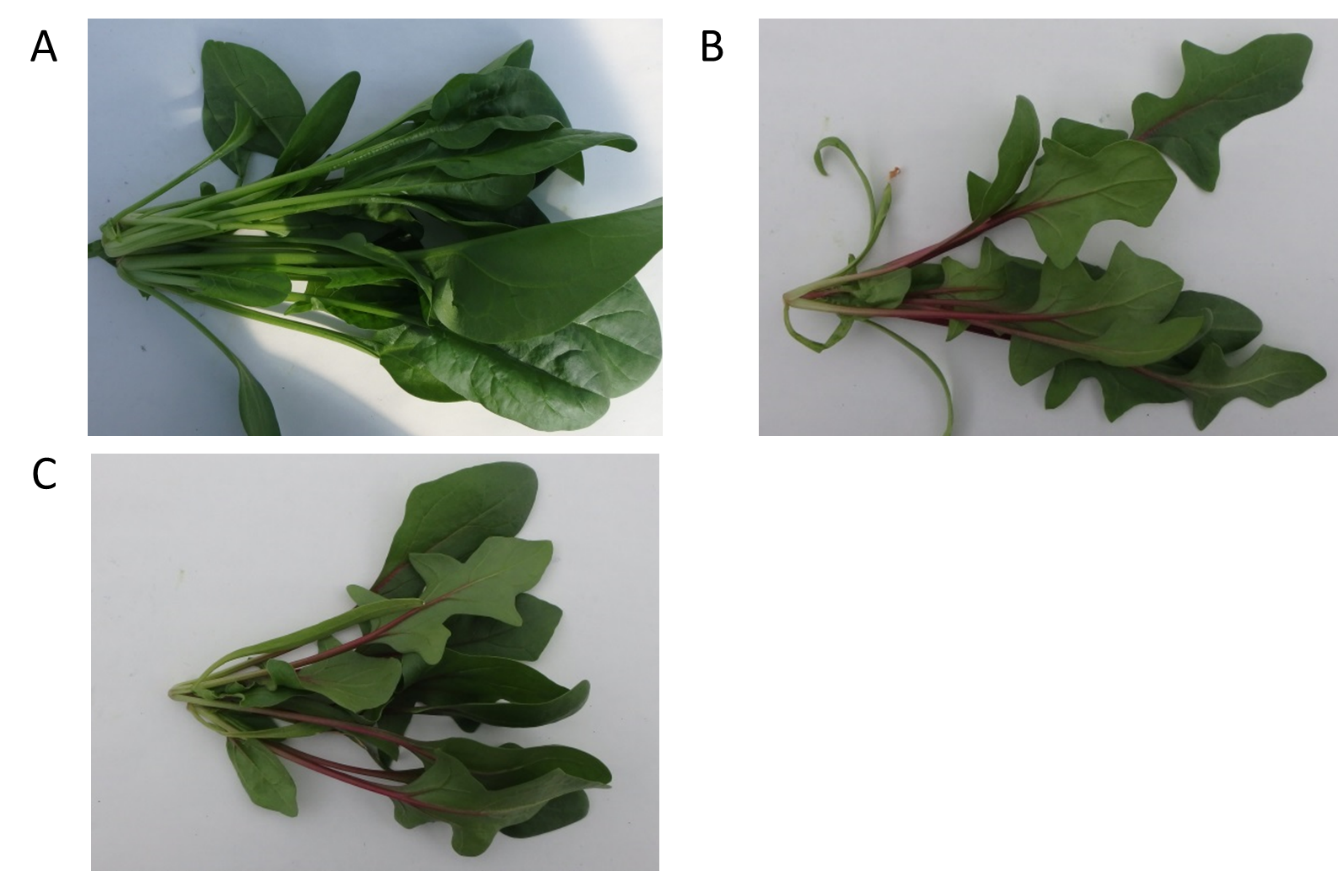


**S1 Fig.** Strains of green- and red-tube spinach (*Spinacia oleracea*) used in this research. (A) NPL08; (B) Sosei salad akari; (C) Banshu akakuki minster.
