# Supplementary figures and images for: Detection of betacyanin in red-tube spinach (Spinacia oleracea) and its biofortification by strategic hydroponics
Source: PLoS One. 2018 Sep 7;13(9):e0203656. doi: 10.1371/journal.pone.0203656 (PMC6128657; doi:10.1371/journal.pone.0203656)

**
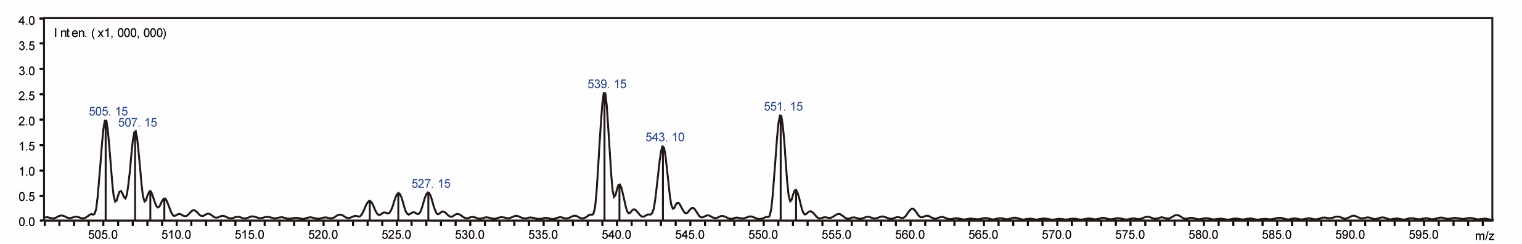
**

**S2 Fig.** Mass spectra of betanin (m/z: 551.15) obtained by LC–MS.

Supplement: S2 Fig — (DOCX) [file pone.0203656.s002.docx]
