# Supplementary material for: Detection of betacyanin in red-tube spinach (Spinacia oleracea) and its biofortification by strategic hydroponics
Source: PLoS One. 2018 Sep 7;13(9):e0203656. doi: 10.1371/journal.pone.0203656 (PMC6128657; doi:10.1371/journal.pone.0203656)
Supplement: S3 Fig — Red-tube spinach were cultured in dopamine-, Ca2+-, and sucrose-added solutions following the schedule shown in Fig 2A. Bars represent the means ± standard deviations of three biological replicates. The statistical significance of differences between the compound-added samples and the control samples (no addition of compounds) was determined by Dunnett’s test (* p < 0.05). Minster, Banchu akakuki minster. (DOCX) [file pone.0203656.s003.docx]

**
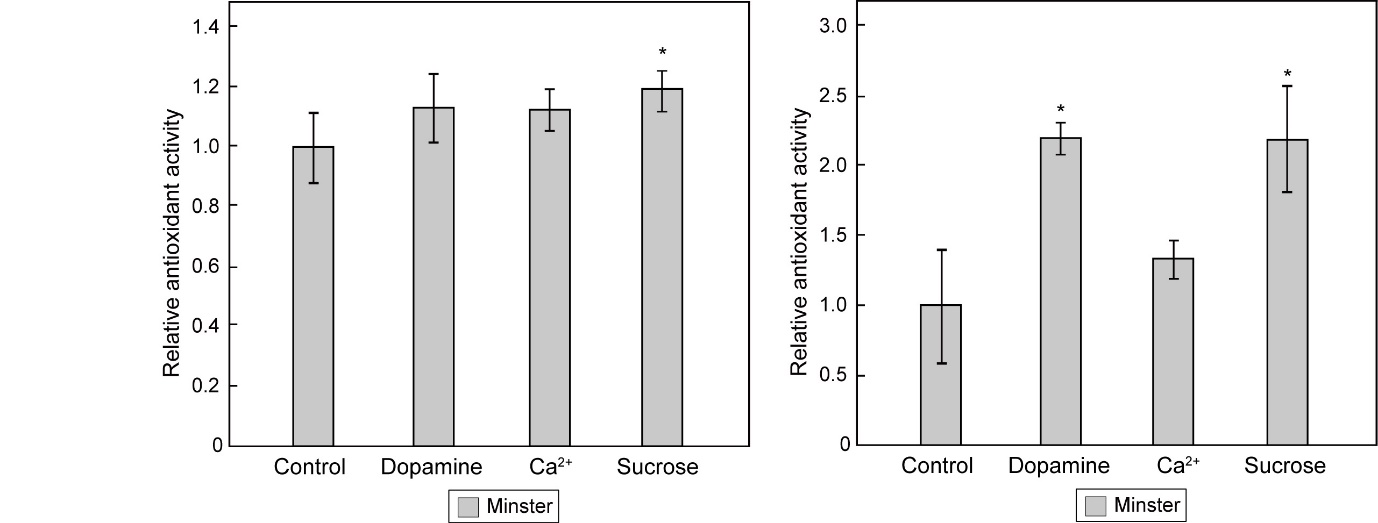
**

**S3 Fig.** Assessment of the antioxidant activity of red-tube spinach (*Spinacia oleracea*) grown in different compound-added solutions. Red-tube spinach were cultured in dopamine-, Ca^2+^-, and sucrose-added solutions following the schedule shown in Figure 2A. Bars represent the means ± standard deviations of three biological replicates. The statistical significance of differences between the compound-added samples and the control samples (no addition of compounds) was determined by Dunnett’s test (* p < 0.05). Minster, Banchu akakuki minster.
