# Supplementary material for: Detection of betacyanin in red-tube spinach (Spinacia oleracea) and its biofortification by strategic hydroponics
Source: PLoS One. 2018 Sep 7;13(9):e0203656. doi: 10.1371/journal.pone.0203656 (PMC6128657; doi:10.1371/journal.pone.0203656)
Supplement: S1 Table — (DOCX) [file pone.0203656.s004.docx]

**S1 Table.** pH of the fertilizer during the hydroponic cultivation.

| **Date** | **Operation** | **pH** | | | |
| --- | --- | --- | --- | --- | --- |
|  |  | **Control** | **Dopamine** | **Ca^2+^** | **Sucrose** |
| **0** | **Planting** | 6.09 | 6.08 | 6.10 | 6.04 |
| **1** |  | 6.14 | 6.17 | 6.16 | 6.21 |
| **2** |  | 6.13 | 6.13 | 6.18 | 6.15 |
| **3** |  | 6.20 | 6.13 | 6.15 | 6.13 |
| **4** |  | 6.06 | 6.00 | 6.07 | 6.03 |
| **5** |  | 5.92 | 5.63 | 6.53 | 5.65 |
| **7** | **First addition** | 5.97 | 5.65 | 6.48 | 6.86 |
| **10** | **Second addition** | 5.78 | 4.71 | 6.27 | 7.28 |
| **11** | **Sampling** | 5.92 | 5.23 | 6.64 | 7.78 |
