# Supplementary material for: Detection of betacyanin in red-tube spinach (Spinacia oleracea) and its biofortification by strategic hydroponics
Source: PLoS One. 2018 Sep 7;13(9):e0203656. doi: 10.1371/journal.pone.0203656 (PMC6128657; doi:10.1371/journal.pone.0203656)
Supplement: S2 Table — (DOCX) [file pone.0203656.s005.docx]

**S2 Table.** Primers used in this study.

| Primer | Sequence (from 5’ to 3’) |
| --- | --- |
| GAPDH_f | CGTAGCTGACTTTTCTGGATTACG |
| GAPDH_r | GCAGCTATGACATCATGAAATGAAG |
| Tyr_f | AAGTCACTCCCAATGAGCGA |
| Tyr_r | CGTGGACCTCAAGTCGAGTA |
| 4,5-DOPA_f | ATAATTTGGGTCGGGCGTTG |
| 4,5-DOPA_r | ATCCAAAGGGTGTGTTGCAC |
| 5GT_f | GGCCTGGAATGATCCACAAC |
| 5GT_r | TTGAACTTGGCTGCACACTC |
| cDOPA_f | GCTGATGGGTCCGATGATTG |
| cDOPA_r | CGGCTCAATTTCCTCCACAG |
| MYB_f | CAACCGCTCATCCTCTCTCT |
| MYB_r | GGCGGAGTAGTTGGCTAGAT |

f: forward primer; r: reverse primer
